# Exploring data-driven algorithms to identify Lyme disease cases in electronic health records

**DOI:** 10.1371/journal.pone.0349121

**Published:** 2026-08-12

**Authors:** Alexandra M. Linz, Erica Scotty, Jennifer K. Meece, Courtney C. Nawrocki, Kiersten J. Kugeler, Sarah A. Hook, Alison F. Hinckley, Anna M. Schotthoefer

**Affiliations:** 1 Marshfield Clinic Research Institute, Marshfield, Wisconsin, United States of America; 2 Centers for Disease Control and Prevention, Fort Collins, Colorado, United States of America; Texas Tech University Health Sciences Center, UNITED STATES OF AMERICA

## Abstract

A tool capable of quickly and accurately identifying Lyme disease (LD) cases in endemic areas would have powerful applications in both research and public health. Using data from a single health system in an area with high LD incidence, we evaluated the feasibility and accuracy of data-driven algorithms to accurately identify LD cases based on easily extracted, structured data elements that are available in electronic health records (EHR). We used random forest and decision tree algorithms to explore the importance of various data elements and to investigate how different combinations of data elements accurately classified clinical events as either LD cases or non-cases. The data elements explored included patient demographics, International Classification of Diseases, 10^th^ Revision, (ICD) codes, LD laboratory tests and their results, and antibiotic prescription orders. Our best performing algorithm was a manually constructed decision tree that incorporated information from three presence/absence variables: an LD ICD code (A69.2x), a positive LD serology test, and a doxycycline order occurring within 30 days of either of the other two elements, which achieved 82% accuracy, 93% sensitivity, 60% adjusted positive predictive value, 68% specificity, and 91% adjusted negative predictive value. Findings from this exploratory effort suggest that data-driven algorithms based on EHR elements hold promise to identify LD cases when detailed chart reviews are impractical. Further efforts may differentiate algorithms between confirmed and probable LD cases, pediatric and adult populations, and utilize text searching to improve accuracy.

## Introduction

Lyme disease (LD) is a tickborne illness with a significant burden in the Midwest and Northeast regions of the United States. In these regions, LD is primarily caused by infection with *Borrelia burgdorferi* and transmitted by infected *Ixodes scapularis* (“blacklegged”) ticks [1]. During early infection, *B. burgdorferi* can cause fever, *erythema migrans* (EM) (a red, expanding, or bullseye-like rash), and flu-like symptoms. If untreated, *B. burgdorferi* can progress to carditis, arthritis, or neuroborreliosis [2,3]. LD is a nationally notifiable disease in the United States, and the number of cases reported to the Centers for Disease Control and Prevention (CDC) has increased over the last three decades, with annual reports surpassing 30,000 cases since 2008 when the case definition expanded to include probable cases [4]. The elevated volume and manual nature of case investigation led to many high incidence states modifying LD surveillance practices, challenging the interpretation of surveillance data at the national level. Consequently, CDC began exploring other data sources to supplement information on the epidemiology of LD [1].

Efforts to examine insurance claims data for identifying LD cases have primarily used *a priori* LD case definitions based on International Classification of Diseases (ICD) codes for LD and LD-appropriate antibiotic prescription orders within a certain time period of LD diagnoses. Findings using these case definitions closely replicate the demographic, seasonal, and geographic trends of LD cases observed in national surveillance data [5–7]. However, diagnosis codes are intended mainly for medical billing, and use of codes for diagnoses may not be wholly accurate or verifiable [8].

A large volume of clinical, laboratory, and potential exposure data is available in electronic health records (EHR) [9,10]. However, it is impractical to manually review all charts in an EHR to identify LD cases. Efforts to apply the LD case definitions used in claims data to EHRs have found that the definitions perform moderately well, with sensitivities and positive predictive values (PPVs) generally > 80%, for patients that meet confirmed, probable, and suspected LD case criteria. However, the definitions did not perform as well at identifying only confirmed or probable cases, with PPVs generally <60% [9–12]. These results suggest that additional research is needed to improve accuracy of these often used *a priori* criteria.

Here, we explored the feasibility and accuracy of a data-driven approach to identify LD cases among those clinically evaluated for LD within an EHR. For this pilot effort, we approached the identification of LD cases as a binary classification problem, with case status assigned as incident LD case or non-case based on standardized manual chart review. We used random forests, a machine learning (ML) method, to sift through EHR-derived variables and gain insight into which variables (alone or in combination) might be most predictive of LD case status [13]. Because random forests can be prone to prediction errors based on unique characteristics, noise, or irrelevant patterns in their training data [14], we also explored development of a decision tree as an alternate method. Our goal was to identify an easily implementable algorithm with at least 70% accuracy for further investigation and refinement across health care systems.

## Methods

### Study population and cohort design

Our study population was identified among patients seeking care during the period 2016–2019 at the Marshfield Clinic Health System in Wisconsin, a geographic region that reports a high burden of LD. The health system served a largely rural population of approximately 750,000 unique patients during the study period. We constructed a cohort that consisted of 67,289 possible clinically-attended LD case events among 56,086 unique patients as described in Kugeler, et al. [12]. Briefly, cohort membership was dependent on the presence of at least one of the following LD-related data elements in the patient’s medical record during the study period: LD ICD code (A69.2x), LD laboratory test order, or LD appropriate 1^st^ or 2^nd^ line antibiotic order. To be included, the latter also had to occur within 30 days of the other two possible data elements or a LD keyword in clinical notes. To account for the possibility that there may be repeat visits associated with a single LD illness, when patients had events with overlapping 30-day windows, we combined them into one event. Therefore, every event in our analysis, even those determined to be non-LD cases, contained at least one of these LD-related elements. Events varied in the combinations of elements present and with regards to the specific details of the elements present, such as by lab test and result, or by antibiotic name, dose, and duration [12]. We assumed that all patients with LD in this health system would fall into this cohort, and its creation enabled more powerful and granular analyses to differentiate between patients with LD and those clinically evaluated but deemed not to have LD.

### Chart review procedure and selection of events for the training, validation, and testing datasets

This research was deemed exempt from full review by the Institutional Review Board of the Marshfield Clinic Research Institute. Patient charts were accessed and analyzed for research purposes between 12/01/2020-09/30/2025. Medical history numbers were used as identifiers to connect chart review results with the multiple data sources used during analysis.

A total of 800 events were selected for manual chart review to determine LD case status for model development and testing. We stratified the selection of events for chart review by seven inclusion criteria groups described in Kugeler, et al. [12] and by LD laboratory test result (any test positive vs. negative) (S1 Table in S2 File). LD case status on chart review was based only on the clinical signs and symptoms associated with the event, without considering LD laboratory tests. This allowed us to evaluate the LD laboratory test-related variables as possible predictors. We used the clinical criteria described in the National Notifiable Diseases Surveillance System’s 2017 LD case definition [15] to assign LD case status of the chart-reviewed events. We defined a LD case as one presenting with: 1) a skin lesion described as EM or using related terms, such as bull’s eye rash, an expanding red, erythematous, or annular rash, or as a rash with central clearing, or 2) signs or symptoms consistent with clinical disseminated or late-stage LD, including new or recently recurring joint pain or swelling, facial palsy, or atrioventricular conduction defects. Patients without symptoms or who had evidence of an alternate diagnosis, such as a positive laboratory test for a different infection, an injury, or evidence to support a connective tissue, cardiac, or neurologic disease other than LD, were deemed non-cases. We created an uncertain LD case classification category that included patients presenting only with non-specific symptoms such as fever, fatigue, headache, myalgia, arthralgia, paresthesia, or neck stiffness. These events (n = 90), along with 246 others that were either missing notes or that were found to be related to a previous LD diagnosis or a different tick-borne disease, were not included in the model development. Our goal was to explore data-driven algorithms that performed best at classifying LD cases and non-cases and then examine how these algorithms classified the uncertain cases to inform future refinement.

There were 5 patients who had two separate clinical events each that were included in the 800 selected for chart reviews. To ensure independence of observations, we randomly selected one event from each of these patients to retain. In total, we included 459 events from unique patients in our initial dataset; of these, 193 (42%) were labeled as LD cases and 266 (58%) were non-cases. We randomly selected two-thirds of each of the LD cases and non-cases for the training dataset (n = 305) and reserved the remaining one-third of cases (n = 154) as our validation dataset. The training dataset was used to select variables and generate the best fitted random forests models. The validation dataset was used to refine the random forest models and fine-tune parameter estimates.

We also generated a testing dataset to assess model performance by randomly selected new events from the same cohort identified by Kugeler et al. (S1 Table in S2 File), excluding patients present in the training and testing datasets and again stratifying by the seven inclusion criteria groups for LD and LD laboratory test results [12,16]. We also stratified this selection by two patient age categories, 0–10 years old and ≥11 years old, as initial results from the training/validation datasets suggested that different data elements were important for the youngest patients. We manually chart reviewed 336 new events and labeled them as LD cases, non-cases, or uncertain cases in the same manner used for the training/validation datasets. The resulting testing dataset was composed of 108 LD (32%) cases and 77 (23%) non-cases. The remaining 151 (45%) events reviewed were deemed uncertain presenting only with non-specific symptoms, having missing notes, or related to possible past rather than incident LD. These uncertain cases were set aside and not included in evaluation of the models; however, the classification of these uncertain cases by the models was examined separately.

### Curation of predictor variables

The variables we investigated as potential predictors included presence/absence of LD ICD codes, LD-appropriate antibiotic prescription orders, LD laboratory test orders, patient age categories, antibiotic drug name, strength, and form, and laboratory test results. Prescription data required standardization of drug names, formulations, and doses as part of the curation process (S2 Table in S2 File). We also included presence/absence variables derived from other ICD diagnosis codes associated with events as potential predictors. To reduce the number and potential collinearity of these ICD codes, we mapped the codes to Phecodes, which group ICD codes into clinically meaningful phenotype groups [17]. We then further grouped the Phecodes into those that might indicate a potential LD symptom and those that would indicate a diagnosis other than LD, such as pyogenic arthritis or pneumonia, as exclusionary diagnoses (S2 Table in S2 File). Because data were electronically pulled from a Research Data Warehouse that combines all diagnosis, laboratory testing, and prescription data, and then verified and corrected with data found by chart review, the absence of data elements in our training/validation and testing sets indicates these elements were not assigned or ordered during the events, rather than missing data. When notes, lab records, or prescription order records were missing from chart review, and we were unable to determine the status of the case for those reasons, we labeled them as such and combined them into the uncertain LD category. All data for the selected records were compiled and curated in R and RStudio using the package “tidyverse” [18–20].

### Algorithm design and model evaluation

We first examined the potential utility of the presence or absence of a LD ICD code, any LD laboratory test, a positive standard two-tier testing (STTT) LD serologic test order or result [21], or an LD-appropriate prescription order using 2 x 2 contingency tables. We also examined the performance of the presence/absence of a doxycycline order because this drug is the preferred 1st-line antibiotic for LD treatment [22] and the most frequently prescribed antibiotic in our datasets (59% overall).

We implemented the random forest model using the R package “randomForest” to examine how combinations of variables performed and to identify a model that optimized accuracy of classifying LD cases and non-cases [23,24]. Eight curated variables were included as possible inputs in model training and validation, including categorical variables for patient age group (0–10, 11–18, 19–64, ≥ 65), drug name, drug strength, STTT result, and presence/absence variables for a LD ICD code, LD symptom Phecode, and exclusionary diagnosis Phecode. The variable for STTT result included a third category for “untested” in addition to “positive” or “negative,” effectively incorporating information about test ordering. We assessed the importance of each variable using the mean decrease in Gini impurity and by measuring the change in accuracy when each variable was ablated from the model [23,24]. The final random forest model iteration included variables that had either greater than 15 mean decrease in Gini impurity or greater than 10% drop in accuracy with ablation in either the full dataset or the 0–10 age group only. Because LD diagnosis and treatment practices differ for children and adults, we ran each model separately on data for the two patient age groups: 0–10 years old and ≥11 years old. The random forest models were built based on 500 trees and by specifying the random selection of three variables at each node. Final model performance was assessed using precision-recall (e.g., PPV-sensitivity) area under curve (PR-AUC), implemented with the R package “prROC” [25].

To construct a more easily interpretable algorithm, we manually built a decision tree in R, informed by the percentages of LD cases and non-cases observed in the categories of the curated variables and the results of random forest model performance. Variable overlap was also considered in deciding which variables to include in the decision tree and in which order.

Performance of the random forest algorithms and decision trees was measured using the following metrics: accuracy (proportion [%] of events correctly classified), positive predictive value [PPV] (also known as precision, which is the proportion [%] of events predicted to be LD cases that were LD cases), sensitivity (also known as recall, which is the proportion [%] of LD cases correctly predicted to be LD cases by the model), specificity (the proportion [%] of non-cases predicted to be non-cases), and the negative predictive value ([NPV]; the proportion [%] of events predicted to be non-cases that were non-cases). We calculated 95% confidence intervals around each proportion. We additionally calculated adjusted PPV and NPV to account for any potential bias induced by our stratified selection methods for chart review. Specifically, we adjusted PPV and NPV by weighting estimates for each inclusion group by the proportion of events in the initial cohort from which events were selected for chart review (S1 Table in S2 File). We further adjusted for the stratified sampling done by age groups (0–10 and ≥ 11 years old) in the testing set and to account for the differing proportions of the groups across the datasets. PPV and NPV were calculated for the age and inclusion group combinations, multiplied by that stratum’s weight, and summed across all strata. Combined, this weighting approach adjusts for bias in selection of charts to reviews so that resulting PPV values are accurate for the entire cohort, which presumably reflects the subset of patients in our health system who were clinically evaluated for LD.

## Results

### Distribution of variables among positive and negative LD cases and the datasets

Patient demographics were similar across the datasets, though there were slightly more females than males in the training set compared to the validation and testing sets. Because we stratified event sampling for the testing dataset by age group, there was a higher proportion of patients in the 0–10 year-old age group in the testing set (Table 1).

**Table 1:** Patient demographics as recorded in the electronic health record in the training, testing, and validation sets.

		Training set (n = 305)	Validation set (n = 154)	Testing set (n = 185)
Sex	Male	147 (48%)	81 (52%)	110 (59%)
	Female	158 (52%)	73 (47%)	75 (41%)
Race	American Indian or Alaska Native	5 (1%)	1 (1%)	1 (1%)
	Asian	1 (<1%)	2 (1%)	0 (0%)
	Native Hawaiian or Other Pacific Islander	0 (0%)	1 (1%)	0 (0%)
	Black or African American	0 (0%)	0 (0%)	0 (0%)
	White	278 (91%)	140 (91%)	173 (94%)
	More than One Race	3 (1%)	2 (1%)	3 (2%)
	Unknown/Not Reported	18 (6%)	8 (52%)	8 (4%)
Ethnicity	Not Hispanic or Latino	286 (94%)	138 (90%)	173 (94%)
	Hispanic or Latino	1 (<1%)	4 (3%)	6 (3%)
	Unknown/Not reported	18 (6%)	12 (8%)	6 (3%)
Age (years)	0-10	31 (10%)	19 (12%)	94 (51%)
	11-18	21 (7%)	6 (4%)	5 (3%)
	19-64	154 (51%)	85 (55%)	48 (26%)
	65+	99 (32%)	44 (29%)	38 (21%)

Consistently across the datasets, 65% of LD cases had a LD ICD code, whereas 19% of non-cases had a code (Table 5). The most common ICD code used for LD was A69.20 (“LD, unspecified”) (88%), with A69.22 (“other neurologic disorders in LD”), A69.23 (“arthritis due to LD), and A69.29 (“other conditions associated with LD”) used rarely (2%, 7%, and 4%, respectively). STTT was performed for >50% of both LD cases and non-cases, though a higher proportion of positive results were observed among the LD cases (Table 2). Doxycycline prescriptions were associated with 74% and 72% of LD cases in the training and validation datasets, respectively, but only 41% of LD cases in the testing dataset (Table 2). This is likely due to the higher proportion of young patients in the testing dataset, who, as is recommended, were more commonly prescribed amoxicillin for LD treatment [22]. Among prescriptions for doxycycline, there was not much variation in the drug strength (95% of the orders for had a strength of 100 mg), whereas the strengths of amoxicillin prescriptions, often adjusted for patient weight, were more variable (S3 Table in S2 File).

**Table 2:** Distribution of variables among Lyme disease cases (LD+) and non-cases (LD-) cases in each dataset used as part of model development. For definitions of exclusionary ICD codes and LD symptom ICD codes, refer to S2 Table in S2 File.

	Chart review designation	Training set (n = 305; LD cases = 128, non-cases = 177)	Validation set (n = 154; LD cases = 65, non-cases = 89)	Testing set (n = 185; LD cases = 108, non-cases = 77)
Has LD ICD code	LD case	86 (67%)	41 (63%)	70 (65%)
	non-case	35 (20%)	16 (18%)	13 (17%)
Standard two-tier testing (STTT) ordered	LD case	81 (63%)	40 (62%)	67 (62%)
	non-case	99 (56%)	49 (55%)	44 (57%)
STTT positive	LD case	25 (20%)	14 (22%)	35 (32%)
	non-case	20 (11%)	5 (6%)	5 (6%)
Prescribed doxycycline	LD case	95 (74%)	47 (72%)	44 (41%)
	non-case	65 (37%)	22 (25%)	11 (14%)
Has exclusionary ICD code	LD case	7 (5%)	4 (6%)	5 (5%)
	non-case	25 (14%)	16 (18%)	14 (18%)
Has LD symptom ICD code	LD case	45 (35%)	27 (42%)	38 (34%)
	non-case	31 (17%)	16 (18%)	25 (31%)

### Univariate assessments of LD-related variables

Presence of a LD ICD diagnosis code had the best univariate performance, correctly classifying 74% of cases, with an adjusted PPV of 72% and specificity of 81%. It performed well for both the 0–10 and ≥ 11-year-old age groups (Table 3). Presence of a LD laboratory test on its own was a poor predictor of LD case status, as tests were not performed for 38% of the LD cases and 56% of non-cases across the combined datasets. Additionally, only 31% of the LD cases across the datasets had positive STTT results, leading to low sensitivity. However, a positive STTT result had the highest specificity (> 90%) observed of any predictor (Table 3).

**Table 3:** Univariate performance of Lyme disease (LD)-related EHR variables independently in classifying LD case status. Displayed are the performance measures (%) for the complete dataset of cases (n = 644), followed by the range of performance values observed across the training, validation, and testing datasets in brackets. For metric names that differ between the fields of ML and epidemiology, we have included the ML term in parentheses. The numbers of cases included in the training, validation, and testing datasets and for the age groups can be found in Table 1.

Variable	Accuracy (%) *correct classifications/ total classifications*	Positive predictive value (%) (Precision) *correct positives/predicted positives*	Sensitivity (%) (Recall) *correct positives/true positives*	Specificity (%) *true negatives/ predicted negatives*	Negative Predictive Value (%) *true negatives/ (true negatives + false negatives)*	Adjusted Positive Predictive Value (%)	Adjusted Negative Predictive Value
**All ages**							
LD ICD	74 [72-75]	75 [71-84]	65 [63-67]	81 [80-83]	73 [63-77]	72 [70-80]	86 [78-88]
LD lab	53 [52-54]	49 [44-60]	62 [62-63]	44 [43-45]	57 [45-62]	25 [15-42]	84 [81-86]
STTT pos	60[58-64]	71[56-74]	25 [20-32]	91 [89-94]	58[50-62]	49 [30-64]	85 [80-86]
Doxycycline order	67 [59-74]	65 [60-80]	62 [41-74]	71 [63-86]	68 [51-79]	54 [49-63]	90 [84-92]
1^st^ or 2^nd^ line treatment order	56 [55-58]	52 [48-62]	83 [69-94]	33 [29-42]	69 [48-88]	37 [34-48]	91 [86-94]
**Ages ≥11 years**							
LD ICD only	72 [66-74]	69 [68-72]	61 [55-65]	80 [77-83]	74 [62-77]	74 [67-76]	82[78-89]
LD lab only	51 [49-52]	45 [43-51]	65 [60-67]	42 [39-42]	62 [47-66]	23 [17-41]	84 [81-85]
STTT pos only	62[58-67]	64[54-76]	22[19-28]	91 [89-95]	62 [54-65]	45 [33-61]	85 [79-86]
Doxycycline order only	73 [70-79]	64 [59-76]	82 [74-85]	66 [60-75]	84 [73-88]	54 [49-62]	92 [85-95]
1^st^ or 2^nd^ line treatment order only	58 [56-65]	50 [48-63]	90 [77-94]	35 [32-52]	82 [68-90]	43 [40-56]	93 [86-96]
**Ages 0–10 years**							
LD ICD only	81 [79-94]	91 [83-94]	77 [72-83]	87 [75-91]	69 [64-86]	93 [84-98]	85 [70-99]
LD lab only	57 [52-59]	69 [64-71]	57 [39-64]	57 [48-75]	44 [42-50]	56 [4-72]	85 [71-86]
STTT pos only	53 [42-57]	87 [50-96]	30 [9-36]	93 [85-97]	44 [41-45]	89 [14-100]	84 [60-90]
Doxycycline order only	45 [45-48]	13 [11-15]	13 [11-15]	98 [88-100]	40 [39-45]	94 [50-100]	78 [64-90]
1^st^ or 2^nd^ line treatment order only	50 [45-58]	69 [62-91]	69 [62-91]	19 [0-27]	26 [0-50]	19 [12-22]	66 [0-71]

When considering patients of all ages and ages ≥ 11 years old, presence/absence of a doxycycline order performed better than presence/absence of any 1^st^ or 2^nd^ line LD antibiotic order, especially with regards to PPV and specificity; sensitivity, however, was higher when a 1^st^ or 2^nd^ line antibiotic order was considered. For patients 0–10 years of age, presence/absence of any 1^st^ or 2^nd^ line LD antibiotic performed better than presence/absence of a doxycycline order; the latter performed poorly, as only 12% of these patients with an antibiotic order had an order for doxycycline across all datasets (Table 3).

### Random forest results

When the random forests included all eight curated variables, accuracies of the models ranged between 69 and 84% for the validation set and 66 and 79% for the testing set. Sensitivities and adjusted PPVs for the models were 60–67% and 53–63%, respectively. Performance metrics were generally highest when the model was run on patients in the 0–10 year old group (Table 4). The results of the mean decrease in Gini impurity and feature ablation identified presence/absence of a LD ICD code as the most important predictor of LD case status, followed by drug strength (S4 Table in S2 File). Because we saw little variation in drug strength, particularly for doxycycline prescriptions (S3 Table in S2 File), we removed drug strength from the model causing drug name to become the second most predictive variable. Phecode variables and STTT results were among the least important (S4 Table in S2 File). When the random forests were run retaining this reduced set of the four most important variables on the testing dataset, we generally observed slight or no improvements in the performance metrics (Table 4).

**Table 4:** Performance of random forest algorithms. We report model performance metrics, % [95% confidence intervals], for the validation and testing datasets. For metric names that differ between the fields of ML and epidemiology, we have included the ML terms in parentheses.

Validation set results	Model formula	Accuracy *correct classifications/ total classifications*	Positive predictive value (Precision) *correct positives/predicted positives*	Sensitivity (Recall) *correct positives/true positives*	Specificity *true negatives/ predicted negatives*	Negative Predictive Value *true negatives/ (true negatives + false negatives)*	Prevalence adjusted positive predictive value	Prevalence-adjusted negative predictive value
All events (n = 154)	LD status ~ all variables	70 [63-77]	66 [59-74]	60 [52-68]	73 [66-80]	78 [71-84]	53 [45-61]	88 [92-93]
Age ≥ 11 (n = 135)	LD status ~ all variables	69 [61-77]	62 [54-70]	57 [49-66]	73 [65-80]	77 [69-84]	49 [40-57]	86 [81-92]
Age 0–10 (n = 19)	LD status ~ all variables	84 [68-100]	83 [67-100]	91 [78-100]	86 [70-100]	75 [56-94]	84 [68-100]	99 [95-100]
All events	LD status ~ Drug name + LD ICD + Symptom Phecodes + Excl. Phecodes	85 [79-91]	88 [82-93]	75 [69-82]	84 [78-90]	92 [88-96]	83 [77-89]	91 [87-96]
Age ≥ 11	LD status ~ Drug name + LD ICD + Symptom Phecodes + Excl. Phecodes	66 [56-76]	71 [62-80]	57 [47-68]	62 [52-72]	75 [66-84]	86 [81-92]	90 [85-95]
Age 0–10	LD status ~ Drug name + LD ICD + Symptom Phecodes + Excl. Phecodes	84 [68-100]	83 [67-100]	91 [78-100]	86 [70-100]	75 [56-94]	84 [68-100]	99 [95-100]
**Testing set results**								
All events (n = 185)	LD status ~ all variables	70 [63-76]	78 [72-84]	67 [54-73]	61 [54-68]	74 [68-80]	63 [55-70]	79 [73-85]
Age ≥ 11 (n = 91)	LD status ~ all variables	66 [56-76]	71 [62-80]	57 [47-68]	62 [52-72]	75 [66-84]	69 [60-79]	79 [70-87]
Age 0–10 (n = 94)	LD status ~ all variables	79 [70-87]	94 [87-99]	72 [63-81]	64 [54-74]	91 [85-97]	98 [95-100]	85 [77-92]
All events	LD status ~ Drug name + LD ICD + Symptom Phecodes + Excl. Phecodes	70 [64-77]	80 [74-86]	66 [59-73]	61 [54-68]	77 [71-83]	29 [23-36]	74 [68-80]
Age ≥ 11	LD status ~ Drug name + LD ICD + Symptom Phecodes + Excl. Phecodes	62 [52-72]	65 [55-75]	55 [45-66]	68 [59-78]	68 [59-78]	25 [16-34]	73 [64-82]
Age 0–10	LD status ~ Drug name + LD ICD + Symptom Phecodes + Excl. Phecodes	80 [72-88]	85 [78-92]	84 [76-91]	71 [61-80]	73 [64-82]	46 [36-56]	89 [83-95]

*”all variables” included were LD ICD code (presence/absence), Lab order(presence/absence), drug name, drug strength, age group category, STTT result, presence/absence of Phecodes indicating a potential Lyme symptom, and presence/absence of Phecodes indicating a potential alternative, exclusionary diagnosis. STTT result was a three category variable including “positive,” “negative,” and “untested.”

We examined characteristics of the false positives and false negatives to understand how the predictive ability of the random forest algorithms failed. Of false negatives, 97% lacked an LD ICD code in their EHR but often had a doxycycline prescription order (49%) or positive laboratory test result (46%). For false positives, 72% had LD ICD codes and 11% had positive STTT results, though 89% lacked a prescription order.

### Decision tree results

We manually built and evaluated a decision tree based on variable importance suggested by the output of our random forest models and univariate analyses (Tables 3, S4 in S2 File). In the decision tree, events were evaluated and classified as LD cases if they satisfied the defined condition at the node; only those that did not meet the condition were passed on to the next node for further evaluation and potential classification. Because the final tree was built manually and not trained, the method did not qualify as a machine learning approach.

**Fig 1:**
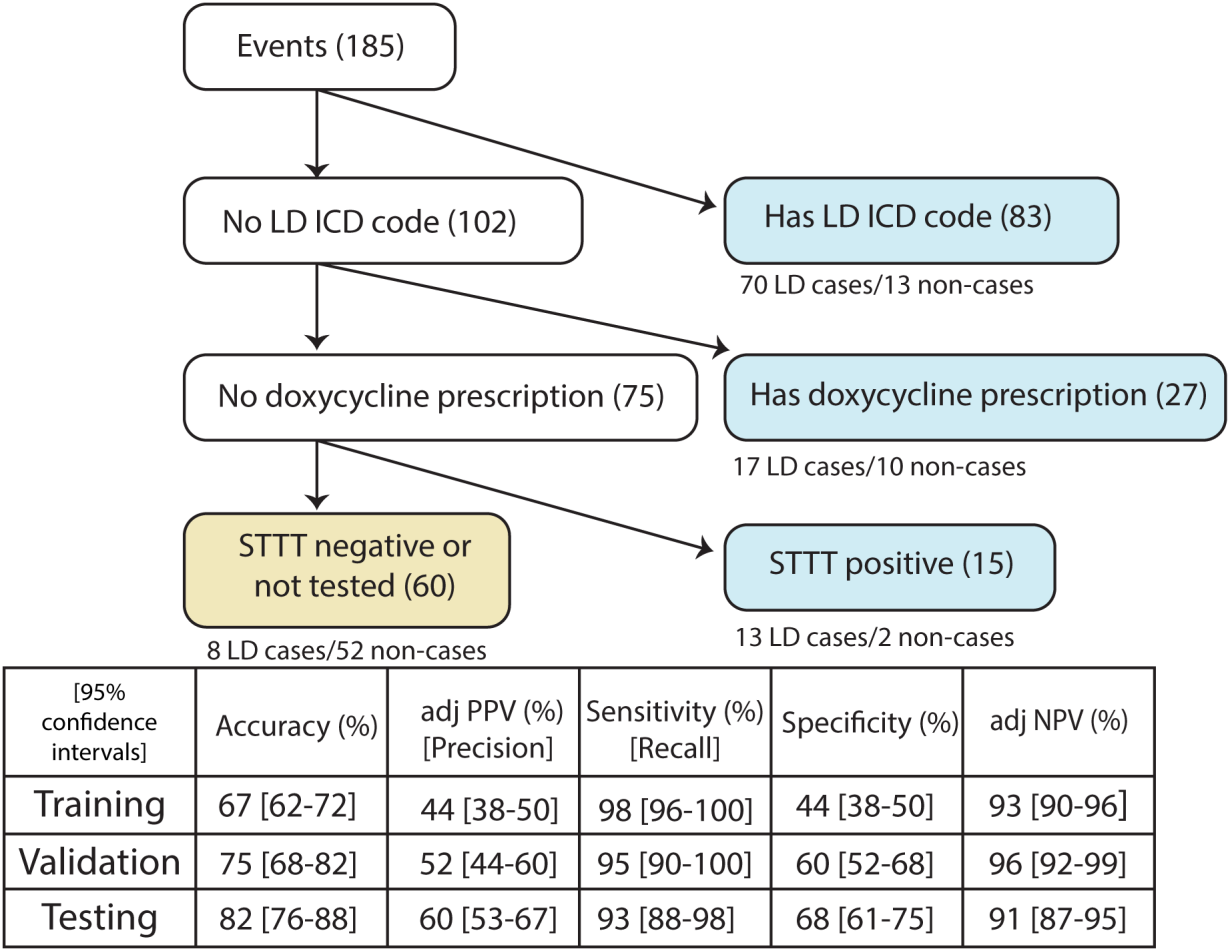
The decision tree manually constructed from the variables identified with the highest rankings of importance in the random forests. Displayed are the branching results from the tree performed on the testing dataset. The numbers of events included in the evaluation at each node are shown in parentheses. The decisions resulting in classification of events at each terminal node are shown as LD cases in blue and as non-cases in yellow. The numbers of LD cases and non-cases classified at each terminal node are displayed under the nodes. Performance measurements (%, [95% confidence intervals]) for the decision tree performed on the training, validation, and testing sets are displayed in the table. Reported values for PPV and NPV were adjusted by strata inclusion to account for differences between datasets.

Our final tree had three branching nodes consisting of the presence/absence of a LD ICD code, presence/absence of doxycycline order (in the absence of a LD ICD code), and presence/absence of a positive STTT result (in the absence of a LD ICD code and doxycycline order) in the event (Fig 1). Attempts to add a fourth node based on the presence/absence of LD symptom or exclusionary Phecodes reduced performance. As such, this tree can be reduced to a heuristic, where the presence of an LD ICD code, doxycycline prescription, or positive STTT result predicts a LD case. If there was no LD ICD code or doxycycline prescription and STTT was negative or not performed, the event was classified as a non-case. The accuracy of this rule for the testing dataset was 82%, above our threshold of 70% (Fig 1). The adjusted PPV and NPV were 60% [CI: 53–67%] and 91% [CI: 87–95%], respectively.

When we examined the incorrect predictions made by our heuristic for the testing set, we observed a false positive rate of 13% and a false negative rate of 4%. Of the 25 total false positive cases, 13 (52%) occurred at the first node, consisting of non-cases with a LD ICD code; 10 (40%) were observed at the second node, with no LD ICD code but with a doxycycline prescription, and the remaining 2 (8%) had no LD ICD code or doxycycline prescription but they did have a positive STTT result. The highest rate of false positives was observed at the doxycycline prescription node, with 10 of the 27 (37%) non-case events incorrectly classified by the tree as LD cases at the node. The 8 LD cases that were incorrectly classified as non-cases did not have a LD ICD code, doxycycline prescription, or positive STTT results. Most of these cases were prescribed amoxicillin or cefuroxime (75%) for LD treatment, and they tended to be in the 0–10 age group (63%).

### Classification of Uncertain LD cases

We explored how the heuristic would classify events that were deemed to be uncertain on chart reviews. These included patients that presented with non-specific signs and symptoms (n = 59), had evidence of a past or history of LD diagnosis (n = 37), had chronic symptoms reported to be related to LD (n = 6), or were missing notes or other information to be able to determine LD status (n = 49). The decision tree predicted 80% of these events to be LD cases (S5 Table in S2 File).

## Discussion

We explored two approaches, random forest ML models and a manually constructed decision tree, to create data-driven algorithms for identifying LD cases in EHRs. We evaluated, alone and in combination, easily extracted variables that have been the basis of *a priori* LD definitions previously used to identify LD cases in insurance claims data and EHRs: LD ICD codes, LD appropriate antibiotic orders, and LD laboratory test data [5–7,9,12]. Additionally, we explored the potential use of other ICD diagnosis codes, combined into LD symptom and exclusionary diagnosis Phecode groupings. Our best performing algorithm, the decision tree, was reduced to a heuristic with cases and non-cases defined by a simple rule that classified events as LD cases when a LD ICD code, prescription order for doxycycline (occurring within 30 days of a LD ICD code or laboratory test), or positive LD serologic test was present. The heuristic had an accuracy of 82% in the testing dataset, above our targeted 70%, and had high sensitivity (>90%).

Our results support the previous *a priori* LD definitions that were based on the presence of a LD ICD code and LD appropriate antibiotic orders co-occurring in time [6,7,9,12,26]; we found that these two variables were the most informative for the correct classification of LD cases and non-cases. Adding information about LD laboratory testing results, when available, also improved accuracy in our heuristic [10]. The presence/absence LD ICD code variable was the most important classifier identified by the random forests, and it had an overall accuracy of 74% when evaluated on its own in our univariate analyses. However, as observed in previous studies, the variable was frequently associated with erroneous predictions, suggesting a possible overreliance on this variable. It has been noted that use of the codes in isolation will inevitably result in missing some true cases. Conversely, many patients that do not meet LD case criteria will receive a LD code in their medical chart, contributing to misclassification [9,27]. Overall, 17–20% of the non-cases included in our datasets had an LD code. We learned from our chart reviews that this often occurred in association with the carry-over of the code from a past LD event to a current event that was unrelated to LD. By adding information about a LD appropriate antibiotic order or a positive LD STTT test result, we were able to boost accuracy, supporting the findings from others that combinations of LD data elements improve classification [9,10,27].

One major challenge in developing an accurate data-driven algorithm is inherent to the difficulty associated with LD diagnosis itself rather than limitations of data elements. The disease can present with a wide spectrum of possible clinical signs and symptoms, and the serologic testing approaches available for laboratory diagnosis have low sensitivity in early stages of disease when most people seek health care for possible LD [3,12,27]. LD serology testing also may remain positive for months to years after infection, which may contribute to overestimation when trying to identify incident cases [28]. Moreover, clinicians may choose to treat for LD even in the absence of confirmatory signs of LD or a positive LD test order. This may make sense for individual patients, as the risks of taking a course of doxycycline may be considered lower than missing an acute LD illness that, if untreated, may disseminate to cause more severe illness [2,3]. However, this practice decreases the utility for a specific treatment order to serve as a proxy for LD case status. Additionally, antibiotics used for LD treatment are used to treat many other infections and conditions, such as upper respiratory infections, pneumonia, urinary tract infections, and otitis media. We attempted to include information about other possible indications for treatment in our algorithms by evaluating the presence/absence of exclusionary Phecodes as a classifier variable; however, this particular variable did not improve accuracy of our models. A previous effort suggested that most LD cases had appropriate prescription drug orders filled within one day of the LD ICD code, so narrowing the time period between prescription orders and other LD-related data in an event may improve algorithm performance [26].

The challenges associated with clinical LD diagnosis and trying to develop an EHR-based algorithm that accurately identifies LD cases are further underscored by the high percentage (80%) of medical charts reviewed and labeled as uncertain LD but that were classified as LD cases by the decision tree. Forty-three percent of these cases had a LD ICD code and 28% had a doxycycline order; however, only 30% had serologic evidence to support a LD diagnosis, suggesting classification of patients with non-specific symptoms or incomplete medical records would be biased, perhaps erroneously, toward LD case classification. Future efforts should explore development of models with more than the two LD case and non-case outcomes we examined here. Outcomes similar to the classification scheme employed in national public health surveillance, which includes categories for confirmed, probable, and suspected cases [15,29], may improve model accuracy by preventing events with conflicting or insufficient information from being labeled as LD cases.

Our findings highlight that a single data-driven algorithm for case identification may not perform equally well for children and adults. Differences in recommended clinical practice, particularly the antibiotics typically used to treat LD in the 0–10 year age group (amoxicillin) compared to older children and adults (doxycycline), drive varying performance of the models [22]. As such, absence of a doxycycline order was the reason many LD cases in the 0–10 year age group were misclassified as false negatives by the random forests and heuristic. We also observed slightly improved performance of the univariate model for the presence/absence of any 1^st^ or 2^nd^ line antibiotic over the presence/absence of a doxycycline order for the younger age group. When we removed the 0–10-year-old age group from the random forest analyses and examined only the cases in the ≥ 11-year-old age group, however, we observed declines in the performance of the models (Table 4). This may be because doxycycline orders for 0–10-year-olds were almost always prescribed for LD treatment, whereas the orders were often prescribed for other indications in adults. Providers also may be more likely to order LD laboratory tests for screening purposes for adults than for children [28,30], such that the combination of a doxycycline order prescribed for a different reason co-occurring with a LD laboratory test in adult non-cases may occur more frequently than for younger children.

The relative proportions of patients aged 0–10 in the various datasets also may have impacted the success of our model development and performance. Our training and validation sets included small sample sizes of young children, which prevented robust training on data from that age group. We tried to compensate for this by stratifying the sampling of our testing set by the two age groups. The resulting pediatric skew in the testing set, compared to the training and validation sets, made it difficult to optimize our heuristic for all age groups because it relied on presence/absence of a doxycycline prescription. Future efforts should better account for the unique characteristics of prescription ordering in young children to determine if different models are needed for children, or if a set of variables exists that would perform best in classifying both children and adults.

The wealth of information available in EHR offers opportunities to explore and develop and refine data-driven algorithms that can ultimately be used to automate LD case identification. This pilot effort highlighted other avenues that should be investigated and could potentially improve accuracy of LD case identification in EHR. Including a step to identify patients with a history of prior LD diagnosis may improve performance, especially those with a positive test result due to prior rather than incident LD. More detailed analyses of ICD codes beyond our groupings into symptom or exclusionary Phecodes may pinpoint specific diagnoses or groups of co-occurring diagnoses to distinguish LD cases and non-cases. Additionally, utilizing clinical text and images in EHR should be explored. For instance, there are ongoing efforts to develop automated tools based on ML and deep learning methods, including convolutional neural networks, capable of differentiating digital images of EM lesions from other lesions and that attempt to generate a probability score that a lesion is EM combining the digital image analysis with patient data reviewed by expert opinion [31–33]. It is possible that such tools may be integrated into LD algorithms in the future as digital images become more widely available in EHRs. ML and deep learning approaches also have been developed to link LD risk to cholesterol levels and social media content [34–36]. As with image data, there may be future opportunities to combine such risk factor associations into EHR-based LD algorithms. Ultimately, different algorithms may be appropriate for surveillance versus research goals. An algorithm optimized for high sensitivity may be desired for purposes of measuring the frequency or incidence of LD diagnoses in a population, whereas an algorithm optimized for high PPV may be desired for identifying cases that should be approached for a clinical trial or other research study.

Here, we found a simple yet highly sensitive heuristic that used structured, easily extracted EHR data elements out-performed random forest models in a single health system. Evaluation and validation of the algorithms are needed across multiple health systems to understand how generalizable this finding is and to identify algorithms that perform well across geographic regions, patient populations, and clinician practices. Further investigation, refinement, and validation of data-driven algorithms are needed to support LD case identification for public health and research purposes.

## Supporting information

S1 File R code used to curate the dataset, run the random forest algorithm, and run the decision tree algorithm. (R)

S2 File Supplemental methods and tables referenced in the text. (DOCX)
