# Supplementary material for: Exploring data-driven algorithms to identify Lyme disease cases in electronic health records
Source: PLoS One. 2026 Aug 12;21(8):e0349121. doi: 10.1371/journal.pone.0349121 (PMC13466019; doi:10.1371/journal.pone.0349121)
Supplement: Supplementary file 1 [file pone.0349121.s002.docx]

**Supplemental Methods, Figures, and Tables**

*Chart review procedure*

Events were included based on the presence of LD-related data elements, including ICD-10 diagnosis codes, laboratory test orders (during the study period, the primary test available was standard two-tiered testing serology (STTT) involving a screening EIA or IFA followed by confirmatory IgM and IgG immunoblots [CDC, 1995]), and prescription orders recommended as first- and second-line treatment for LD (doxycycline, amoxicillin, cefuroxime, amoxicillin-clavulanate, azithromycin, cefotaxime, ceftriaxone, clarithromycin, erythromycin, and penicillin G). These orders had to have a duration of at least seven days, a prescription date within 30 days of another LD data element or LD-related term in a clinical note, and no explicit text-based instructions for a non-LD indication (11). We defined events as starting with the earliest LD data elements and searching for other LD data elements within 30 days. This 30-day window advanced from each LD data element until no more were found. Multiple events per patient were allowed, as long as there were more than 30 days between LD data elements.

**Table S1. Distribution of reviewed LD medical charts among the seven combinations of LD data elements present in events selected for inclusion in the training/validation and testing datasets.**

| Inclusion group | Definition | Training/validation dataset  n = 459 | Testing dataset  n = 185 |
| --- | --- | --- | --- |
| **1** | Lyme ICD + laboratory testing + prescription | 108 (24%) | 33 (18%) |
| **2** | Lyme ICD + prescription | 74 (16%) | 30 (16%) |
| **3** | Laboratory testing + prescription | 82 (18%) | 24 (13%) |
| **4** | Lyme ICD only | 17 (4%) | 12 (6%) |
| **5** | Laboratory testing only | 48 (10%) | 24 (13%) |
| **6** | Lyme ICD + laboratory testing | 31 (7%) | 30 (16%) |
| **7** | Prescription only | 99 (22%) | 32 (17%) |

*ICD code conversion and aggregation*

In total, we identified 1,170 non-LD ICD codes present, and those grouped into 363 Phecodes. Phecodes for common chronic health issues such as hypertension, hyperlipidemia, gastroesophageal reflux disease, diabetes, hypothyroidism, and obesity were removed from consideration in the analysis. Of those remaining and that occurred more than 5 times in the training set, we further grouped into two binary categorical variables, one as a “LD symptom Phecodes,” category for those Phecodes that might be indicative of an acute LD symptom and the second as an “exclusionary Phecodes” category for those that might indicate a diagnosis other than LD (Table 3). ICD code to Phecode conversions are available at <https://phenomics.va.ornl.gov/phecodemap/>. These “symptom” and “exclusionary” Phecodes variables were included in the datasets as possible presence/absence predictors.

**Table S2. Most common Phecodes associated with potential Lyme encounters and their groupings into “LD symptom” and “exclusionary” categories.**

| **Phecode** | **Categorical designation** | **Counts in training set (n = 305)** | **Counts in validation set (n = 154)** | **Counts in testing set (n = 185)** |
| --- | --- | --- | --- | --- |
| Rash and other nonspecific skin eruption (687.1) | Potential LD symptom | 28 | 20 | 39 |
| Fever of unknown origin (783) | Potential LD symptom | 26 | 10 | 39 |
| Other headache syndromes (339) | Potential LD symptom | 19 | 11 | 32 |
| Myalgia and myositis unspecified (770) | Potential LD symptom | 12 | 4 | 15 |
| Cough (512.8) | Exclusionary diagnosis | 12 | 4 | 10 |
| Acute pharyngitis (465.2) | Exclusionary diagnosis | 7 | 5 | 9 |
| Urinary tract infection (591) | Exclusionary diagnosis | 5 | 4 | 5 |
| Atopic/contact dermatitis due to other or unspecified (939) | Exclusionary diagnosis | 4 | 5 | 5 |
| Dizziness and giddiness (386.9) | Potential LD symptom | 7 | 4 | 5 |
| Pneumonia (480) | Exclusionary diagnosis | 4 | 5 | 2 |

**Supplemental Results**

**Table S3. Prescription drug data associated with LD cases and non-cases.** Information about the strength in milligrams and the range of observed strengths are included below the number of events for each drug.

| **Number (%)**  **of events Mode [min- max range] strength in mg/day** | **Training dataset (n = 305)** |  | **Validation dataset (n = 154)** |  | **Testing dataset (n = 185)** |  |
| --- | --- | --- | --- | --- | --- | --- |
|  | **LD cases n=128** | **Non-cases n=177** | **LD cases n=65** | **Non-cases n=89** | **LD cases n=108** | **Non-cases n= 77** |
| **Amoxicillin** | 15 (14%)  2000  [500-2000] | 22 (12%)  500  [250-2000] | 9 (14%)  2000  [250-2000] | 8 (9%)  500, 2000  [400-2000] | 25 (23%)  2000  [250-2000] | 20 (26%)  2000  [250-2000] |
| **Amoxicillin-clavulanate** | 0 (0%) | 29 (16%)  875  [875-3000] | 0 (0%) | 27 (30%)  875  [500-3000] | 2 (2%)  2000  [2000] | 8 (10%)  875, 3000  [875-3000] |
| **Azithromycin** | 1 (1%)  1000  [1000] | 3 (2%)  250  [250] | 0 (0%) | 1 (1%)  250  [250] | 0 (0%) | 0 (0%) |
| **Ceftriaxone** | 0 (0%) | 2 (1%)  1000  [1000] | 2 (3%)  1000  [1000] | 0 (0%) | 0 (0%) | 4 (5%)  1000  [1000-2000] |
| **Cefuroxime** | 5 (4%)  500  [500-1250] | 4 (2%)  500  [250-500] | 3 (5%)  500  [500-1250] | 3 (4%)  500  [500] | 3 (3%)  500  [500-1250] | 2 (3%)  500  [500] |
| **Doxycycline** | 95 (74%)  100  [50-100] | 65 (37%)  100  [50-100] | 47 (72%)  100  [100] | 22 (25%)  100  [20-100] | 44 (41%)  100  [50-125] | 11 (14%)  100  [20-100] |

**Table S4. Ranking importance of variables in the random forest model.** These metrics were measured in the model containing all variables. Larger values in the mean decrease in Gini Index indicate a more predictive variable. The drop in accuracy was measured via ablation, ie re-running the model with a single variable removed and re-assessing accuracy in the validation set.

| **Variable** | **Mean Decrease in Gini Index (all events)** | **Mean Decrease in Gini Index (age 0-10 years)** | **Drop in accuracy without variable (all events) (%)** | **Drop in accuracy without variable (age 0-10 years)** |
| --- | --- | --- | --- | --- |
| Lyme ICD code | 33.0 | 6.7 | 26 | 27 |
| Drug strength* | 23.3 | 2.6 | 2 | 6 |
| Drug name* | 12.7 | 2.9 | 1 | 13 |
| Age group | 10.4 | NA | -5 | NA |
| Symptom Phecodes | 9.1 | 0.3 | 1 | 13 |
| Two tier testing result | 6.0 | 1.1 | 3 | 5 |
| Exclusionary Phecodes | 3.1 | 0.6 | 1 | 12 |

*When drug strength is removed, the value for drug name increases to 29.6 for all events and 4.2 for those with age less than or equal to 10. NA= Not applicable.

**Table S5. Predictions of the decision tree on “uncertain” encounters.**

| Reason for uncertainty | Predicted LD case | Predicted non-case |
| --- | --- | --- |
| Past LD diagnosis | 34 | 3 |
| Chronic symptoms potentially associated with LD | 5 | 1 |
| Non-specific signs and symptoms | 45 | 14 |
| Incomplete or missing records | 37 | 12 |
| Total | 121 (80%) | 30 (20%) |

**Figure S1. PR AUC evaluation of random forest models.** The plot on the left was generated by the model containing all variables based on the validation set. The plot on the right was generated using only the variables Lyme ICD code presence/absence, drug name, exclusionary Phecodes, and symptom Phecodes. The areas under the curves were measured at 0.87 for the left and 0.70 for the right.
